# The effects of fixation target size and luminance on microsaccades and square-wave jerks

**DOI:** 10.7717/peerj.9

**Published:** 2013-02-12

**Authors:** Michael B. McCamy, Ali Najafian Jazi, Jorge Otero-Millan, Stephen L. Macknik, Susana Martinez-Conde

**Affiliations:** 1 Department of Neurobiology, Barrow Neurological Institute, USA; 2 Department of Signal Theory and Communications, University of Vigo, Spain; 3 Department of Neurosurgery, Barrow Neurological Institute, USA

**Keywords:** Saccadic intrusions, Fixation control, Fixation error

## Abstract

A large amount of classic and contemporary vision studies require subjects to fixate a target. Target fixation serves as a normalizing factor across studies, promoting the field’s ability to compare and contrast experiments. Yet, fixation target parameters, including luminance, contrast, size, shape and color, vary across studies, potentially affecting the interpretation of results. Previous research on the effects of fixation target size and luminance on the control of fixation position rendered conflicting results, and no study has examined the effects of fixation target characteristics on square-wave jerks, the most common type of saccadic intrusion. Here we set out to determine the effects of fixation target size and luminance on the characteristics of microsaccades and square-wave jerks, over a large range of stimulus parameters. Human subjects fixated a circular target with varying luminance and size while we recorded their eye movements with an infrared video tracker (EyeLink 1000, SR Research). We detected microsaccades and SWJs automatically with objective algorithms developed previously. Microsaccade rates decreased linearly and microsaccade magnitudes increased linearly with target size. The percent of microsaccades forming part of SWJs decreased, and the time from the end of the initial SWJ saccade to the beginning of the second SWJ saccade (SWJ inter-saccadic interval; ISI) increased with target size. The microsaccadic preference for horizontal direction also decreased moderately with target size . Target luminance did not affect significantly microsaccades or SWJs, however. In the absence of a fixation target, microsaccades became scarcer and larger, while SWJ prevalence decreased and SWJ ISIs increased. Thus, the choice of fixation target can affect experimental outcomes, especially in human factors and in visual and oculomotor studies. These results have implications for previous and future research conducted under fixation conditions, and should encourage forthcoming studies to report the size of fixation targets to aid the interpretation and replication of their results.

## Introduction

A large amount of classic and contemporary psychophysical and physiological vision studies require subjects to fixate a target. Target fixation serves as a normalizing factor across studies, promoting the field’s ability to compare and contrast experiments. Yet, there is no standard as to the preferred characteristics of such a target; thus, fixation target parameters, including luminance, contrast, size, shape and color, vary across experiments. Presently, fixation targets are chromatic or achromatic, their sizes range typically from 0.05 to 2 degrees of visual angle (°), and their shapes are as diverse as circles, concentric rings, squares, and crosses (Bonneh et al., 2010; Hsieh & Tse, 2009; Kanai & Kamitani, 2011; Laubrock et al., 2010; McCamy et al., 2012; Mergenthaler & Engbert, 2010; Otero-Millan, Macknik & Martinez-Conde, 2012; Pastukhov & Braun, 2010; Rolfs et al., 2011; Thaler et al., 2012).

Previous studies have investigated the effects of fixation target size and luminance on the control of fixation position, with conflicting results (Rolfs, 2009). The discrepancies across studies could arise from: (a) differences in eye-tracking techniques; (b) use of experienced fixators, often the authors themselves, versus naïve subjects; (c) small subject sample sizes. For instance, Steinman (1965) and Rattle (1969) each studied the effect of target size on the fixation parameters of two subjects, with inconsistent results. Steinman (1965) found conflicting effects of target size on fixation accuracy in the two observers that participated in his study, although larger targets led to fewer microsaccades in both subjects. Rattle (1969) found a modest decrease in fixation accuracy for large targets, and a larger reduction in fixation accuracy for targets the size of the fovea. Thus, the evidence is contradictory as to how target size affects fixation accuracy. Studies on the effects of target luminance on fixation parameters have rendered more consistent results, though few luminance levels have been tested (Steinman, 1965). Here we set out to determine the effects of fixation target size and luminance on the characteristics of microsaccades and square-wave jerks (SWJs; the most common type of saccadic intrusion, consisting of an initial saccade away from the target followed, after a short delay, by a return saccade that brings the eye back onto target), over a large range of stimulus parameters.

Human subjects fixated a circular target with varying luminance and size while we recorded their eye movements non-invasively with a high-speed video tracker. Microsaccade rates decreased linearly and microsaccade magnitudes increased linearly with target size. The percent of microsaccades forming part of SWJs (heretofore SWJ prevalence) decreased, and the time from the end of the initial SWJ saccade to the beginning of the second SWJ saccade (heretofore SWJ inter-saccadic interval; ISI) increased with target size. The microsaccadic preference for horizontal direction also decreased moderately with target size. Target luminance did not affect significantly microsaccades or SWJs. In the absence of a fixation target, microsaccades became scarcer and larger, while SWJ prevalence decreased and SWJ ISIs increased. Thus, the choice of fixation target can affect experimental outcomes, especially in human factors and in visual and oculomotor studies. These results have implications for previous and future research conducted under fixation conditions, and should encourage forthcoming studies to report the size of fixation targets to aid the interpretation and replication of their results; this is not done in every paper (Bonneh et al., 2010; Kanai & Kamitani, 2011; Murakami, 2010).

## Materials and Methods

### Subjects

Seventeen adult subjects (12 male, 5 female) with normal or corrected-to-normal vision participated in the experiment. Age and education information was not obtained. Sixteen naive subjects were paid $15/session and one subject was an author (MBM). Experiments were carried out under the guidelines of the Barrow Neurological Institute’s Institutional Review Board (protocol number 04BN039). Written informed consent was obtained from each subject.

### Experimental design

Subjects rested their forehead and chin on the EyeLink 1000 head/chin support 57 cm away from a linearized video monitor (Barco Reference Calibrator V, 75 Hz refresh rate). Subjects were instructed to look at the center of a circular target presented on the center of the monitor’s screen, on a 50% gray background. Target luminance and size varied randomly across trials. Eleven possible luminance levels (ranging from 5% to 95% in 9% steps) and six possible radius sizes (0.033°, 0.067°, 0.133°, 0.267°, 0.533°, and 1.067°) resulted in a total of 66 experimental conditions. Note that, for a luminance level of 50%, there was no fixation target, and in this case the subjects were instructed to look at the center of the monitor. The experiment consisted of 4 sessions of ∼30 min, with each session including 33 randomly interleaved trials of 30 s each. Each subject saw each fixation target twice (i.e. 60 s of presentation time for each visible fixation target condition) and the no fixation target condition 12 times (i.e. 360 s: 6 sizes at 50% luminance, with each size seen twice). Subjects took short (∼2–5 min) breaks after each 11 trials. Subjects’ eye position was calibrated at the beginning of the experimental session, and re-calibrated after each break. We used custom code and the Psychophysics Toolbox (Brainard, 1997; Kleiner et al., 2007; Pelli, 1997) to display visual stimuli. To disregard the potential effect of the initial stimulus onset transient at the start of each trial, we conducted analyses only on data recorded after the first second of the trial.

### Eye movement analyses

**Figure 1:**
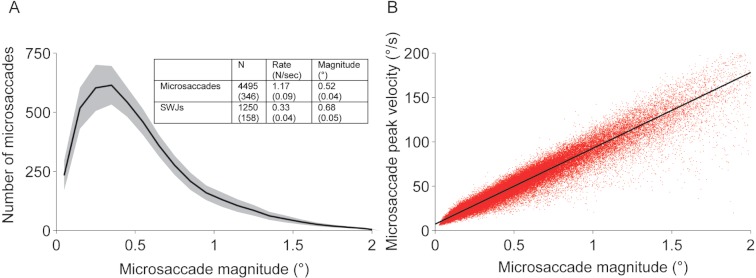
Microsaccades and SWJs. (A) Average microsaccade magnitude distribution across subjects and experimental conditions. Inset: Microsaccade and SWJ descriptive statistics. Shadow and numbers in parentheses indicate the s.e.m. across subjects (*n* = 17). (B) Microsaccadic peak velocity-magnitude relationship for all subjects combined. Each red dot represents a microsaccade.

**Figure 2:**
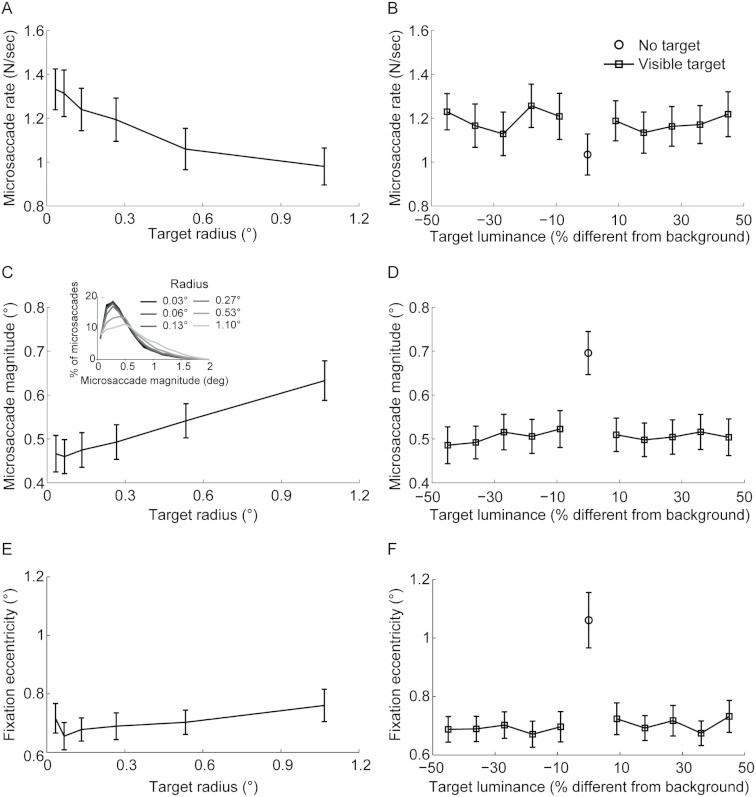
Target size but not luminance affects microsaccade rate, magnitude, and fixation eccentricity. (A) Microsaccade rate decreased linearly with target size (*F*(5,80) = 20.24,*p* < 0.0001; linear trend *F*(1,16) = 16.00,*p* < 0.0001). (B) Microsaccade rate did not change significantly with target luminance (*F*(9,144) = 2.14,*p* = 0.082). (C) Microsaccade magnitude increased linearly with target size (*F*(5,80) = 28.96,*p* < 0.0001; linear trend *F*(1,16) = 39.20,*p* < 0.0001). (D) Microsaccade magnitude did not change significantly with target luminance (*F*(9,144) = 1.6,*p* = 0.121). (E) Fixation eccentricity increased linearly with target size (*F*(5,80) = 5.82,*p* < 0.001; linear trend *F*(1,16) = 9.71,*p* = 0.007). (F) Fixation eccentricity did not change significantly with target luminance (*F*(9,144) = 1.16,*p* = 0.326). Microsaccade rate (B) was lower, whereas microsaccade magnitude (D) and fixation eccentricity (F) were higher, when the fixation target was absent compared to present (rate: *t*(16) = 2.96,*p* = 0.009; magnitude: *t*(16) = −5.64,*p* < 0.001; fixation eccentricity: *t*(16) = −5.94,*p* < 0.0001). Error bars represent the s.e.m. across subjects (*n* = 17).

Binocular eye position was acquired noninvasively with a video eye tracker at 500 Hz (EyeLink 1000, SR Research, instrument noise 0.01° RMS). We identified and removed blinks as portions of the raw data where pupil information was missing. We also removed portions of data where very fast decreases and increases in pupil area occurred (>50 units/sample, such periods are probably semi-blinks where the pupil is never fully occluded) (McCamy et al., 2012; Troncoso et al., 2008). We added 200 ms before and after each blink/semi-blink to eliminate the initial and final parts where the pupil was still partially occluded (Troncoso et al., 2008). Saccades were identified with a modified version of the algorithm developed by Engbert and Kliegl (Engbert, 2006; Engbert & Kliegl, 2003; Engbert & Mergenthaler, 2006; Laubrock, Engbert & Kliegl, 2005; Rolfs, Laubrock & Kliegl, 2006) with λ = 4 (used for the velocity threshold detection) and a minimum saccadic duration of 6 ms. To reduce the amount of potential noise, we considered only binocular saccades, that is, saccades with a minimum overlap of one data sample in both eyes (Engbert, 2006; Engbert & Mergenthaler, 2006; Laubrock, Engbert & Kliegl, 2005; Rolfs, Laubrock & Kliegl, 2006). Some saccades are followed by a fast and small saccadic eye movement in the opposite direction, called dynamic overshoot, which is often more prominent in the eye that moves in the abducting direction (Kapoula, Robinson & Hain, 1986). Unlike the return saccade in a SWJ, a dynamic overshoot follows a saccade without latency between the two movements. We identified dynamic overshoots as saccades that occurred less than 20 ms after a preceding saccade (Møller et al., 2002) and did not consider them as new saccades. Microsaccades were defined as saccades with magnitude <2° in each eye (Beer, Heckel & Greenlee, 2008; Betta & Turatto, 2006; Hafed, Goffart & Krauzlis, 2009; Martinez-Conde et al., 2006; Martinez-Conde et al., 2009; Troncoso et al., 2008). This threshold was selected to accommodate the shift to increased microsaccade magnitudes that occurred with larger target sizes (Fig. 2C **Inset**). When calculating microsaccade properties such as magnitude, peak velocity, and direction we averaged the values for the right and left eyes. See Fig. 1 for microsaccade descriptive statistics and the microsaccadic main sequence (peak-velocity relationship).

We defined a SWJ as the combination of one small saccade that moves the eye away from the fixation target, followed after a short period by a second corrective saccade directed back towards the target (Abadi & Gowen, 2004; Leigh & Zee, 2006; Martinez-Conde, 2006; Otero-Millan et al., 2011a). We identified SWJs using the algorithm developed in Otero-Millan et al. (2011b). This method measures how similar a given saccade pair (that is, a pair of consecutive saccades) is to an ideal SWJ. In an “ideal SWJ” the two saccades are separated by a short interval (usually around 200 ms), have the same magnitudes, and their directions are exactly opposite (Otero-Millan et al., 2011b). We calculated a SWJ index based on the these three defining SWJ characteristics: (a) the direction dissimilarity of first and second saccade, (b) the magnitude similarity of first and second saccade, and (c) the temporal proximity of first and second saccade. The SWJ index provides a single, continuous variable between zero and one for each saccade pair. Values closer to one indicate more similarity to an ideal SWJ. If a saccade pair’s SWJ index was larger than a given threshold (Otero-Millan et al., 2011b), we classified the pair as a SWJ. We defined SWJ magnitude as the average magnitude of the two saccades defining the SWJ, for the eye that the SWJ occurred in (SWJs are not necessarily conjugate, though most are Herishanu & Sharpe (1981)). Thus, we averaged the magnitudes of all SWJs in each eye, and then averaged across eyes (Fig. 1A). Similarly, we calculated SWJ ISIs by determining the average SWJ ISI for each eye, and then averaging across eyes.

### Statistical methods

To assess the effects of target luminance and size on microsaccades and SWJs, we performed a repeated measures ANOVA on each of the dependent variables: microsaccade rate, microsaccade magnitude, SWJ magnitude, SWJ ISI, SWJ prevalence, the deviation of microsaccade direction from horizontal, and fixation eccentricity (i.e. gaze distance to the center of the target, or to the center of the screen if no target was present) after a microsaccade. Target luminance (10 levels, we excluded the luminance level of 50% because it matched the background luminance, thus the target was invisible) and size (6 levels) were the within subjects factors variables. For violations of the ANOVA assumption of sphericity, *p*-values were adjusted using the Greenhouse-Geisser correction. To compare microsaccade characteristics with vs. without a fixation target, we performed two tailed paired *t*-tests on the same dependent variables. In this case, we collapsed all fixation target conditions with luminance ≠ 50% for the fixation target condition; a fixation target with luminance = 50% corresponded to the no fixation target condition. Significance levels were set at α = 0.05 throughout.

## Results

### Effects of target size and luminance

Microsaccade rate decreased linearly with target size, whereas microsaccade magnitude and fixation eccentricity increased linearly (Figs. 2A, 2C, 2E). Target luminance did not affect microsaccade rate, magnitude, or fixation eccentricity (Figs. 2B, 2D, 2F). There was no interaction between target luminance and size for any of these variables (all *F*-values <1.4).

Our data show for the first time that microsaccade magnitude increases with target size. Steinman (1965) previously found that larger fixation targets lead to fewer microsaccades – consistent with our present results – but did not investigate whether target size had an effect on microsaccade magnitude. The lack of effects of target luminance on microsaccade rate is consistent with previous results (Steinman, 1965).

**Figure 3:**
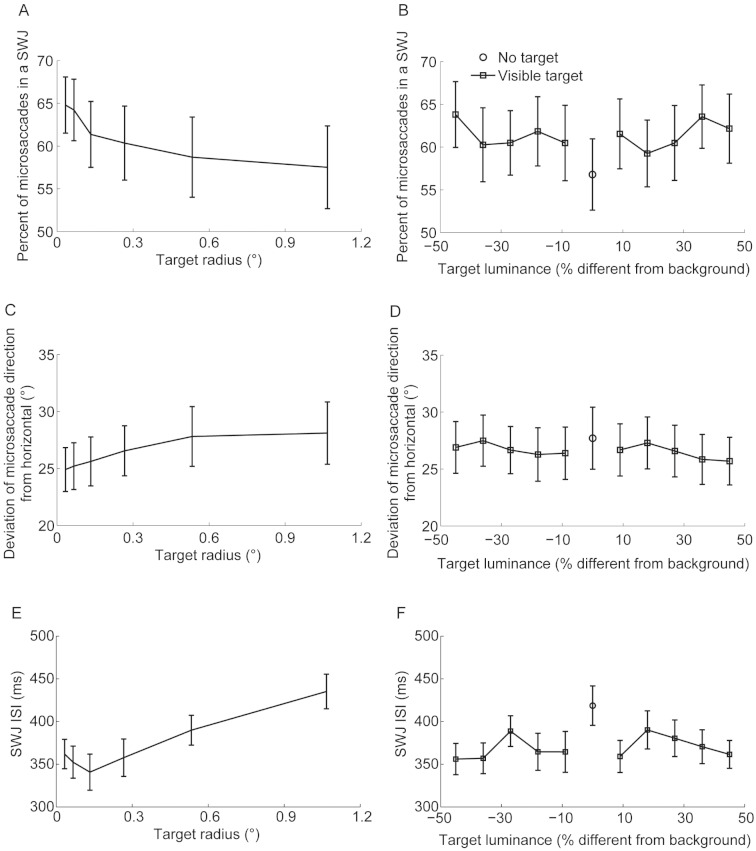
Target size but not luminance affects microsaccade direction, SWJ prevalence, and SWJ ISIs. (A) SWJ prevalence decreased linearly with target size (*F*(5,80) = 5.83,*p* = 0.005; linear trend *F*(1,16) = 11.70,*p* = 0.004). (B) SWJ prevalence did not change significantly with target luminance (*F*(9,144) = 1.19,*p* = 0.304). (C) Microsaccadic deviation from horizontal direction increased linearly with target size (*F*(5,80) = 4.87,*p* = 0.013); linear trend *F*(1,16) = 8.81,*p* = 0.009). (D) Microsaccadic deviation from horizontal direction did not change significantly with target luminance (*F*(9,144) = 1.13,*p* = 0.347). (E) SWJ ISIs increased linearly with target size (*F*(5,80) = 16.11,*p* < 0.0001; linear trend *F*(1,16) = 32.79,*p* < 0.0001). (F) SWJ ISIs did not change significantly with target luminance (*F*(9,144) = 1.35,*p* = 0.216). (B) Less microsaccades were part of a SWJ (*t*(16) = 2.29,*p* = 0.036) and (F) SWJ ISIs were higher in the absence than in the presence of a fixation target (*t*(16) = −3.30,*p* = 0.005). (D) The microsaccadic preference for horizontal direction was equivalent in the absence of a fixation target and in the presence of one (*t*(16) = −1.30,*p* = 0.213). Error bars represent the s.e.m. across subjects (*n* = 17).

Otero-Millan et al. found that larger microsaccades are more likely to be part of SWJs (Otero-Millan et al., 2011b). Here we found increased microsaccade magnitudes with bigger targets; thus we had expected to find a higher prevalence of SWJs with greater target sizes. Surprisingly, we found instead a significant linear decrease in the prevalence of SWJs with target size (Fig. 3A). Human SWJs are typically composed of horizontal microsaccades (Abadi & Gowen, 2004; Otero-Millan et al., 2011b). Thus, one possible explanation for the decreased prevalence of SWJs with increased target size could be that microsaccades became less horizontal with target size. Indeed, this turned out to be the case: the vertical component of microsaccade direction increased linearly, although moderately, with target size (Fig. 3C). A SWJ’s return saccade typically corrects the fixation error introduced by the initial SWJ saccade. We found that SWJ ISIs increased linearly with target size, suggesting longer latencies for error detection with larger fixation targets. Target luminance did not affect microsaccade direction, SWJ prevalence (Figs. 3B, 3D), or SWJ ISIs (Figs. 3E–3F). There was no interaction between target luminance and size for microsaccade direction, SWJ prevalence, or SWJ ISIs (all *F*-values <1). Target size affected SWJ rate and magnitude in the same way that it did microsaccade rate and magnitude (data not shown).

### Effects of presence versus absence of a fixation target

We investigated the effect of not having a fixation target on microsaccade parameters during fixation. To do this, we collapsed all the conditions with a fixation target (i.e. target luminance ≠ 50%) and compared them with the condition where there was no target (i.e. target luminance = 50%). Microsaccades were scarcer and larger without a target than with a target (Figs. 2B, 2D). These findings extend and are consistent with, those of a recent report of smaller rates and larger microsaccade magnitudes during attempted fixation of the center of a black screen, compared to attempted fixation of a 0.0667° target with maximum contrast on a black background (Cherici et al., 2012). The present data also agree with the previous observation of lower microsaccade rates during the free-viewing exploration of blank scenes than during that of natural scenes (Otero-Millan et al., 2008). Fixation eccentricity was significantly larger in the absence than in the presence of a target (Fig. 2F).

Again, because SWJs are more likely to occur with larger microsaccades, we had expected to find a higher prevalence of SWJs in the absence of a fixation target. To the contrary, we found once again that microsaccades were less likely to be part of a SWJ in the absence than in the presence of a fixation target (Fig. 3B). In this case however, microsaccades were not significantly less horizontal in the absence than in the presence of a fixation target (Fig. 3D). SWJ ISIs increased without a fixation target, again suggesting delayed detection of fixation errors in the absence of a target (Fig. 3F). The presence or absence of a fixation target affected SWJ rate and magnitude in the same way it did microsaccade rate and magnitude (data not shown).

## Discussion

We found that microsaccade rate and preference for horizontal direction decreased linearly and that microsaccade magnitude increased linearly with fixation target size. No previous research had found an increase in microsaccadic magnitude or a decrease in preference for horizontal direction with target size. We also examined, for the first time, the effects of fixation target characteristics on SWJ parameters, and found that SWJ prevalence decreased linearly with target size, whereas SWJ ISIs increased with target size. Target luminance had no effect on microsaccade or SWJ parameters. In the absence of a fixation target, microsaccade rates decreased and magnitudes increased, whereas SWJ prevalence decreased and SWJ ISIs increased.

A simple theory, similar to that described in Timberlake et al. (1972), may explain the effect of target size on microsaccades and SWJs. When fixation targets are small enough to fit entirely in the fovea, fixation error detection may depend mostly on retinal signals (i.e. visual errors signaled by deviations of the target image from the preferred fixation position (Putnam et al., 2005)), whereas proprioceptive signals may be less important. When the fixation target extends beyond the fovea, where spatial resolution is diminished, proprioceptive signals may play a bigger role in keeping the target in the preferred location. Proprioceptive signals have less spatial/temporal resolution than foveal retinal signals (Hansen & Skavenski, 1977; Van Beers, Sittig & Denier van der Gon, 1998), thus potentially resulting in delayed detection of (larger) fixation errors and decreased production of microsaccades able to correct them. Such microsaccades, when generated, will be necessarily larger than with smaller fixation targets. This hypothesis could also account for the results obtained in the absence of a fixation target, as in that case the entire monitor screen becomes the fixation target (i.e. a very large fixation target). The proposed framework is also compatible with the decreased SWJ prevalence and longer SWJ ISIs we found with larger targets. The second saccade of a SWJ typically corrects a first saccade that takes the subject’s optimal fixation location (Putnam et al., 2005) away from the point of interest (Abadi & Gowen, 2004; Otero-Millan et al., 2011b). If error detection abilities decrease with target size, as discussed above, corrective saccades may occur less frequently and with longer latencies, therefore reducing the instances of square-wave coupling and increasing SWJ ISIs.

Alternatively, the reduced preference for horizontal microsaccade direction with increased target size could explain our results: because SWJ are comprised of mostly horizontal microsaccades (Abadi & Gowen, 2004; Otero-Millan et al., 2011b), larger target sizes could reduce SWJ coupling. The explanatory power of this hypothesis is limited, however, because the effect of target size on microsaccade direction was small.

It is also possible that subjects may have relaxed their fixation somewhat with larger target sizes. For instance, they may have felt that they were accomplishing their task as long as their gaze position remained inside the target (despite having been instructed to look at the center of the target/monitor).

We conclude that future studies requiring subjects to fixate must choose and tune carefully the characteristics of the fixation target to the needs of the experiment. For instance, studies requiring subjects to produce fewer or larger microsaccades may use a larger fixation target, or no fixation target at all. Our results also indicate the need to report the characteristics of fixation targets in future research to aid data interpretation and replication.
